# Comparison of the oxidation of carcinogenic aristolochic acid I and II by microsomal cytochromes P450 in vitro: experimental and theoretical approaches

**DOI:** 10.1007/s00706-017-2014-9

**Published:** 2017-07-26

**Authors:** Václav Martínek, František Bárta, Petr Hodek, Eva Frei, Heinz H. Schmeiser, Volker M. Arlt, Marie Stiborová

**Affiliations:** 1 Department of Biochemistry, Faculty of Science, Charles University, Albertov 2030, 128 40 Prague 2, Czech Republic; 2 Division of Radiopharmaceutical Chemistry, German Cancer Research Center (DKFZ), Im Neuenheimer Feld 280, 69120 Heidelberg, Germany; 3 Analytical and Environmental Sciences Division, MRC-PHE Centre for Environment and Health, King’s College London, London, SE1 9NH UK; 4 NIHR Health Protection Research Unit in Health Impact of Environmental Hazards at King’s College London in Partnership with Public Health England, London, SE1 9NH UK

**Keywords:** Enzymes, Redox reactions, High pressure liquid chromatography, Molecular modeling

## Abstract

**Abstract:**

The herbal drug aristolochic acid, a natural mixture of 8-methoxy-6-nitrophenanthro[3,4-*d*]-1,3-dioxole-5-carboxylic acid (AAI) and 6-nitrophenanthro[3,4-*d*]-1,3-dioxole-5-carboxylic acid (AAII), is derived from *Aristolochia* species and is the cause of two nephropathies. Ingestion of aristolochic acid is associated with the development of urothelial tumors linked with aristolochic acid nephropathy and is implicated in the development of Balkan endemic nephropathy-associated urothelial tumors. The *O*-demethylated metabolite of AAI, 8-hydroxyaristolochic acid (AAIa), is the detoxification product of AAI generated by its oxidative metabolism. Whereas the formation of AAIa from AAI by cytochrome P450 (CYP) enzymes has been found in vitro and in vivo, this metabolite has not been found from AAII as yet. Therefore, the present study has been designed to compare the amenability of AAI and AAII to oxidation; experimental and theoretical approaches were used for such a study. In the case of experimental approaches, the enzyme (CYP)-mediated formation of AAIa from both carcinogens was investigated using CYP enzymes present in subcellular microsomal fractions and recombinant CYP enzymes. We found that in contrast to AAI, AAII is oxidized only by several CYP enzymatic systems and their efficiency is much lower for oxidation of AAII than AAI. Using the theoretical approaches, such as flexible *in silico* docking methods and ab initio calculations, contribution to explanation of these differences was established. Indeed, the results found by both used approaches determined the reasons why AAI is better oxidized than AAII; the key factor causing the differences in AAI and AAII oxidation is their different amenability to chemical oxidation.

**Graphical abstract:**

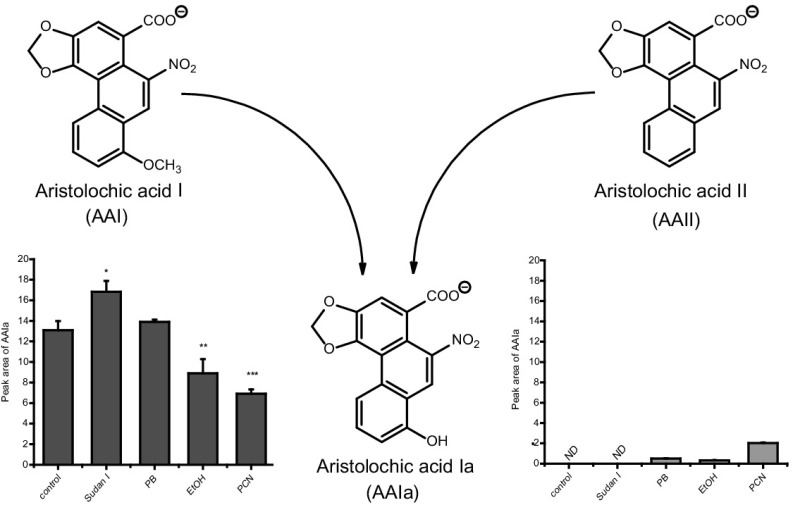

## Introduction

**Fig. 1:**
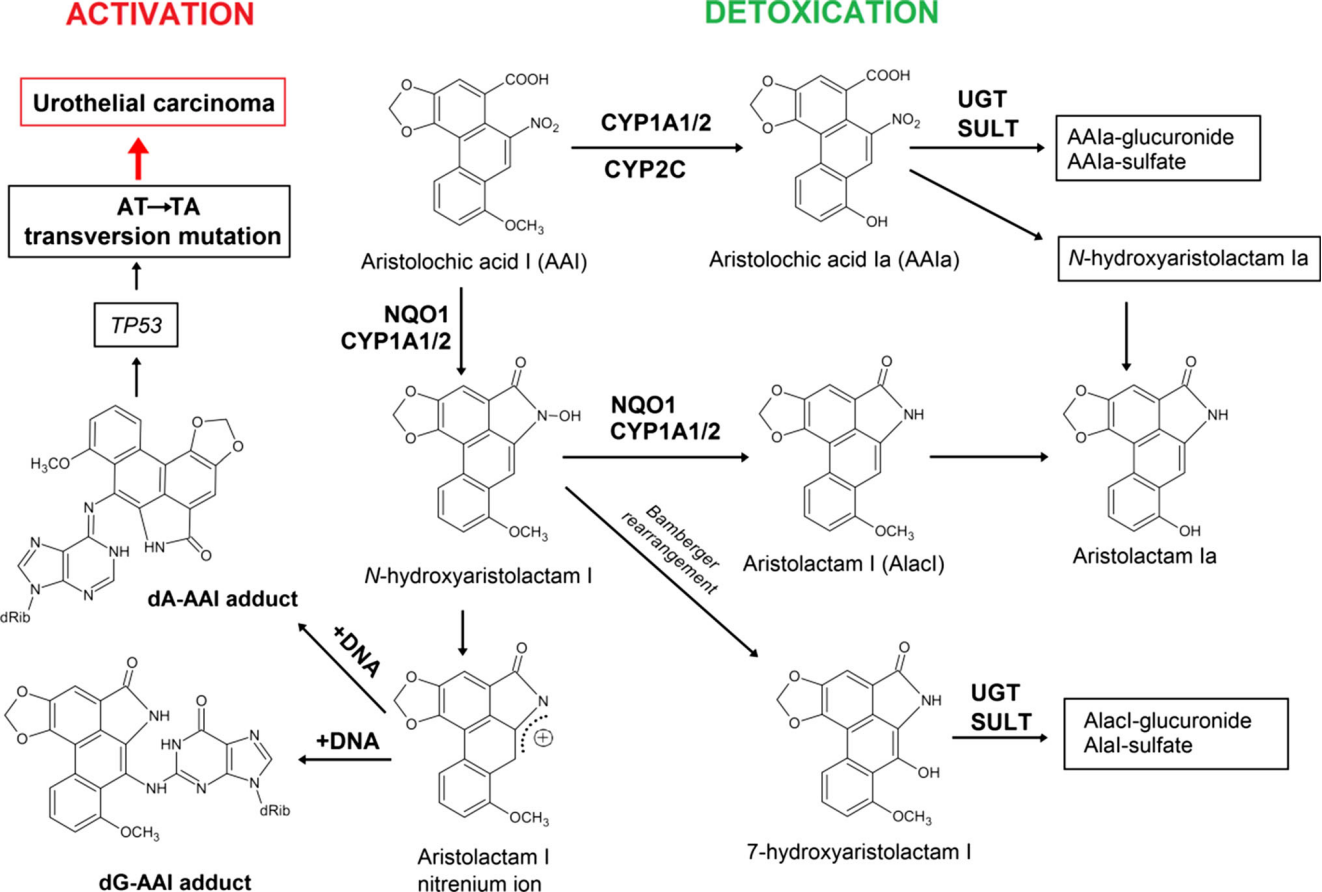
Activation and detoxication pathways of AAI. *dA-AAI* 7-(deoxyadenosin-*N* ^6^-yl)aristolactam I, *dG-AAI* 7-(deoxyguanosin-*N* ^2^-yl)aristolactam I, *CYP1A1/2* cytochrome P450 1A1 and 1A2, *CYP2C* cytochrome P450 2C, *NQO1* NAD(P)H:quinone oxidoreductase, *UGT* UDP glucuronosyltransferase, *SULT* sulfotransferase

Aristolochic acid (AA), the natural extract of plants of the *Aristolochiaceae* family, is a mixture of structurally related nitrophenanthrene carboxylic acids, with two major components aristolochic acid I (8-methoxy-6-nitrophenanthro[3,4-*d*]-1,3-dioxole-5-carboxylic acid, AAI; Fig. 1) and aristolochic acid II (6-nitrophenanthro[3,4-*d*]-1,3-dioxole-5-carboxylic acid, AAII). AA is found exclusively in plants of both the *Aristolochia* and *Asarum* genera of the family *Aristolochiaceae* in all parts. *Aristolochia* herbs have been used for remedies throughout the world since antiquity and they remain in use today, particularly in Chinese herbal medicine [1–9]. Both AAI and AAII are mutagenic and genotoxic [1, 10–13] forming covalent adducts with DNA, the genotoxic end points generated by their reductive activation in vitro and in vivo (Fig. 1) (reviewed in [1–9]). In 2012 AA was classified as carcinogenic to humans (group 1) acting by a genotoxic mechanism by the International Agency for Research on Cancer (IARC) [14]. Today, there is compelling evidence that human exposure to AA leads to chronic renal disease and upper urinary tract cancer known as aristolochic acid nephropathy (AAN) [14, 15], which is now recognized as a global disease [9]. AA is also considered as the major cause of another chronic renal disease associated with urothelial malignancy known as Balkan endemic nephropathy (BEN) [2, 3, 6–9, 15].

The metabolism of AA has been studied in several species demonstrating that the major metabolites found in urine and feces are the aristolactams I and II [16–18] produced by six electron reductions of the nitro group. Other minor metabolites formed through O-demethylation and denitration have also been reported. The only metabolites identified in humans so far are the aristolactams I and II found in urine [16].

On comparing the AAI with AAII, significantly higher levels of AAI-derived DNA adducts than adducts derived from AAII were found in several organs of rats and mice exposed to these compounds in vivo [19–23] and in various enzymatic systems in vitro [24–30]. However, in C3H/He mice exposed to equivalent doses of AAI and AAII, lower levels of AAII-derived DNA adducts were found only in non-target organs such as liver, stomach, intestine and lung, in contrast to the primary target tissues, renal cortex, medulla and bladder (urothelial cells) [22], where the same extent of formation of DNA adducts was found. The apparent discrepancies among the studies [19–23] might be attributed to several reasons such as the use of various animal models, utilization of a variety of treatment protocols and/or employing the different AA–DNA adduct detection methods.

Differences in levels of AAI- and AAII-derived DNA adducts found in most studies performed in vivo and in vitro might be also caused by a different enzymatic conversion of these carcinogens, leading both to their activation to DNA adducts and their detoxification. Indeed, finding that AAII is a poorer substrate of the reduction-catalyzing enzymes located in microsomal and cytosolic subcellular fractions than AAI has been already demonstrated in several studies [25, 28, 29]. However, even though the efficiency of biotransformation enzymes to oxidize AAI was determined, such information is missing for AAII.

It has been shown that the genotoxic and carcinogenic properties caused by AAI- and AAII–DNA adduct formation is mediated by their partial reduction to the reactive acylnitrenium ion, which is a prerequisite for their generation in vivo and in vitro [7, 8]. Several human enzymes capable of activating AA by nitroreduction have been identified and include cytosolic NAD(P)H:quinone oxidoreductase (NQO1) [28–31] and microsomal enzymes, mainly cytochrome P450 (CYP) 1A1 and CYP1A2, while NADPH:CYP oxidoreductase (POR) is less efficient [25, 26, 32–35]. In addition to the abilities of CYP1A1 and 1A2 to reduce AAI and AAII, these enzymes are also efficient in AAI oxidative detoxification to form the *O*-demethylated metabolite, 8-hydroxyaristolochic acid (AAIa) [33, 34, 36–40] (Fig. 1). This dual role of CYP1A1 and 1A2 is an important feature, because a balance between reductive activation and oxidative detoxification reactions of AAI is considered to be a critical determinant in the development of AAN and BEN. However, until the present time the knowledge of the participation of AAII in these processes is scarce, because the oxidative metabolism of AAII is essentially not known; no metabolites formed by direct oxidation of AAII have been identified in human and animal models in vivo [16–18], and no data on metabolism of AAII to its oxidative metabolites in vitro have been reported.

Therefore, the present study is primarily focused on the investigation of the CYP-mediated oxidation of AAII in vitro and secondly on a comparison of AAII-reaction product(s) with those of AAI. To this end, rat and human hepatic microsomal subcellular fractions containing CYPs and recombinant CYP enzymes were used to analyze their potency to catalyze the oxidation of AAI and AAII. Moreover, *in silico* docking, employing soft–soft (flexible) docking procedure, was used to study the interactions of AAI and AAII with the CYP-compounds I, the highly reactive CYP intermediates that are responsible for the CYP-mediated oxidations of their substrates [41, 42], of the most efficient CYP enzymes oxidizing AAI [39]. Furher, ab initio calculations were employed to investigate the amenability of AAI and AAII to oxidation. We believe that such a study might shed more light on mechanisms of the CYP-mediated metabolism to AAI and AAII and their contribution to AAN and BEN development.

## Results and discussion

### Oxidation of AAI and AAII to AAIa by CYP enzymatic systems

To identify the efficiencies of individual CYPs to oxidize AAII, three approaches were employed: (1) the use of rat and human hepatic microsomes, (2) the use of specific inducers of CYP enzymes to modulate the levels of individual CYPs in rat microsomes and (3) the use of rat and human recombinant CYPs.

**Fig. 2:**
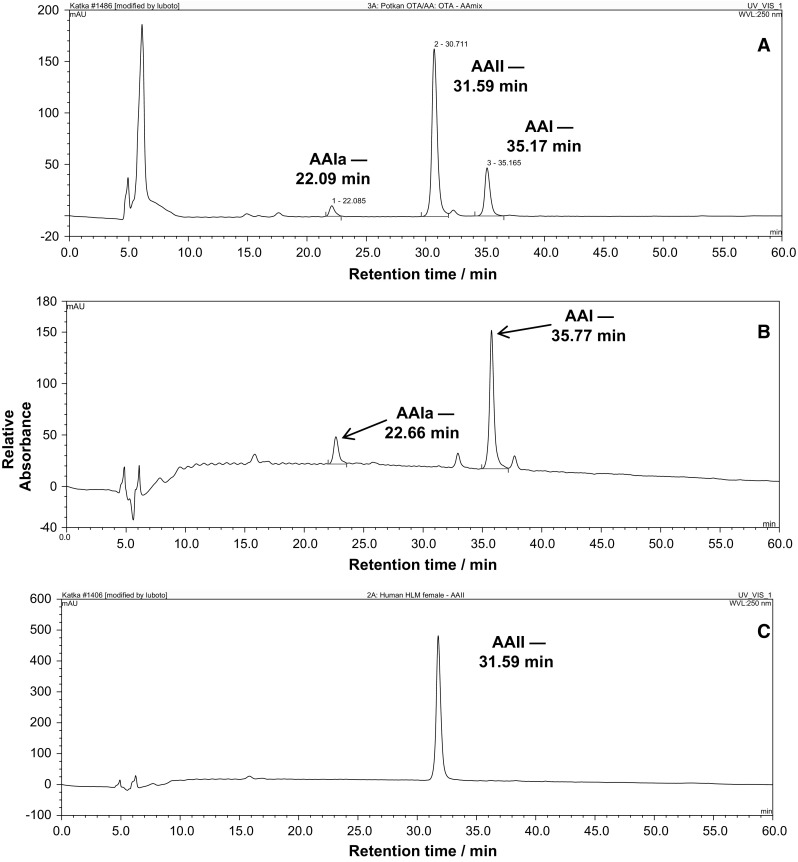
HPLC chromatogram of AA oxidation metabolites formed by hepatic microsomes of control rats incubated with AA and NADPH (**a**), that of AAI oxidation metabolites by the same microsomal fraction incubated with AAI and NADPH (**b**), and AAII metabolites formed by the same microsomal fraction incubated with AAII and NADPH (**c**). HPLC was carried out with a Nucleosil 100-5 C_18_, 25 × 4.0 mm, 5 mm (Macherey-Nagel) column, using a linear gradient of acetonitrile (20–60% acetonitrile in 55 min) in 100 mmol dm^−3^ triethylamonium acetate with a flow rate of 0.6 cm^−3^ min^−1^. A Dionex HPLC pump P580 with UV/VIS UVD 170S/340S spectrophotometer detector set at 254 nm was used. Peaks were integrated with CHROMELEON™ 6.01 integrator. A peak eluting at retention time (r.t.) 22.1 (22.7) min was identified as AAIa using mass spectroscopy analysis [38]

Oxidation of the AA natural mixture, consisting of AAI and AAII as the major components, and that of pure AAI or AAII by rat hepatic microsomes was analyzed by HPLC (Fig. 2). Incubation of AA, where AAI and AAII are present, or AAI alone, with rat microsomes and NADPH (a coenzyme of the CYP-monooxygenase system) leads to the formation of AAIa (Fig. 2a, b).

In contrast, no AAII metabolites were detectable when AAII was incubated with these microsomes under the same conditions; no AAIa or other peak products of AAII were detectable by HPLC under the used conditions (Fig. 2c). Likewise, no AAIa or other peak products of AAII were found when human hepatic microsomes were used (results not shown).

**Fig. 3:**
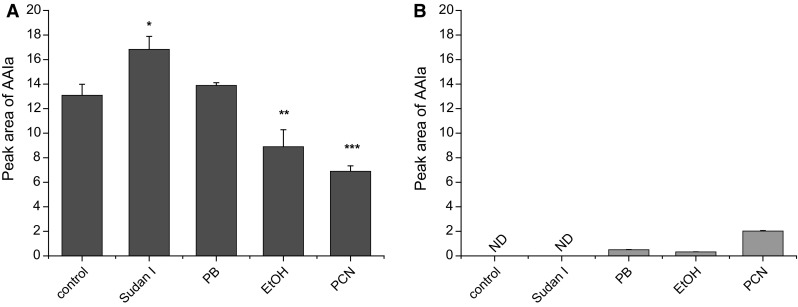
AAIa formation by rat hepatic microsomes from AAI (**a**) [38] and AAII (**b**). Values represent mean ± standard deviations from three independent experiments. ***P < 0.001, **P < 0.01, *P < 0.05 (Student’s *t* test), significantly different from incubations of AAI with control microsomes. *ND* not detectable

To investigate whether AAII might be oxidized to AAIa by rat hepatic microsomes enriched with individual CYP enzymes, we used selective CYP inducers (Fig. 3). Using this approach, we evaluated which of the CYPs can participate in the formation of AAIa from AAI and AAII in these rat hepatic microsomes (Fig. 3). In the case of AAI, as described in our former studies [36, 38], hepatic microsomes isolated from rats treated with Sudan I (as an inducer of CYP1A), phenobarbital (PB) (as an inducer of CYP2B and 2C), ethanol (EtOH) (which induces CYP2E1), and pregnenolone carbonitrile (PCN) (as an inducer of CYP3A) *O*-demethylated AAI to AAIa. The highest level of AAIa was formed by microsomes of rats treated with Sudan I (rich in CYP1A), followed by microsomes of rats treated with PB (rich in CYP2B and 2C) and microsomes of rats untreated—control (Fig. 3a). In contrast to these results, the AAIa formation from AAII was detectable only using microsomes of rats pre-treated with PCN (as an inducer of CYP3A), followed by those with PB (rich in CYP2B and 2C) and those with ethanol (rich in CYP2E1) (Fig. 3b). These microsomes were, however, much less effective to form AAIa from AAII than from AAI.

In our former studies, we examined the activity of individual human and rat CYPs to oxidize AAI to AAIa using recombinant enzymes heterologously expressed in microsomes of baculovirus-infected insect cells (Supersomes™) in combination with their reductase, NADPH:CYP reductase (POR) [33, 38, 39]. In these studies, it was demonstrated that human CYPs were more effective in AAI oxidation than their rat orthologs. Human and rat CYPs of the 1A subfamily are the major enzymes oxidizing AAI. Other CYPs such as human and rat CYPs of the 2C subfamily and human CYP3A (CYP3A4/5), 2D6, 2E1 and 1B1, also form AAIa, but with much lower efficiency than CYP1A (see Fig. 2a in our previous study [39]). Only rat CYP enzymes of the 1A and 2C subfamilies oxidize AAI of which CYP1A enzymes are more active than CYP2C enzymes (see Fig. 2b in the former study [39]).

**Fig. 4:**
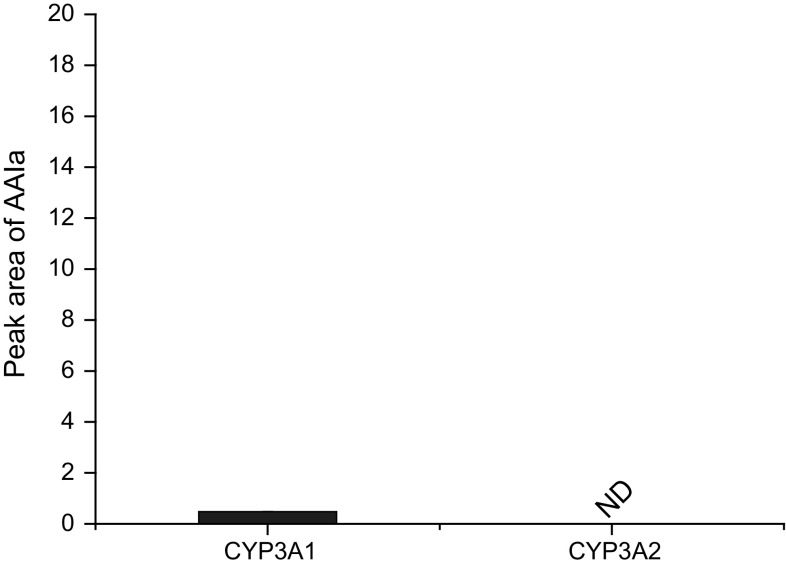
AAIa formation by rat CYP3A1 in Supersomes™. Values represent mean ± standard deviations from three independent experiments

Of human and rat CYP enzymes expressed in the same CYP systems (Supersomes™), only rat CYP of the 3A subfamily, CYP3A1, but not CYP3A2, was capable of oxidizing AAII to AAIa (Fig. 4). Other tested CYPs such as those of CYPs of the 1A, 2B, 2C and 2E subfamilies were inefficient in this reaction (results not shown).

### Origin of differences in CYPs-mediated oxidation between AAI and AAII

Experimentally observed differences in CYPs-mediated oxidation between AAI and AAII were further investigated using a combination of theoretical methods. The efficiency of CYP enzymes, which were able to oxidize AAI to form AAIa in vitro, was much higher than their ability to oxidize AAII (Fig. 3). Several properties of the molecules might be responsible for this variation in their metabolism: (1) different nature of their interaction with the CYP enzymes or (2) different susceptibility of AAI and AAII toward oxidation reaction leading to AAIa metabolite.

### Binding of AAI and AAII to the active sites of CYP1A1, 1A2 and 3A4

**Fig. 5:**
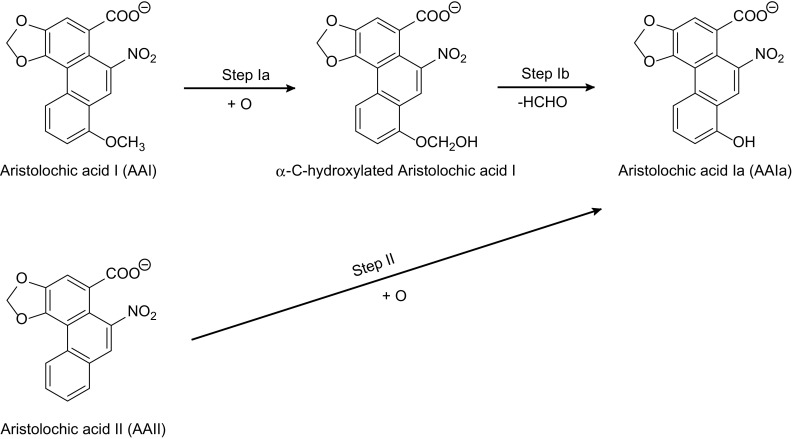
Scheme of AAI and AAII oxidation to AAIa

O-Demethylation of AAI and C8-ring hydroxylation of AAII proceed (Fig. 5) via the CYP-mediated attack of activated oxygen atom of compound I on the target carbons.

The high affinity binding of a substrate (AAI or AAII) is necessary for efficient enzymatic activity; therefore, we used soft–soft, flexible molecular docking to generate and rank possible binding poses of AAI and AAII in active sites of selected CYP enzymes. The substrate orientation in the narrow active site of mentioned enzymes is also important, as only suitable substrate position, allowing sterical contact between reacting groups, facilitate the catalysis. Thus, differences among the abilities of the CYP enzymes to *O*-demethylate AAI or to directly mono-oxygenate carbon 8 of AAII might be caused by the affinities of AAI and AAII to these enzymes and their binding orientations in their active sites.

Previously, we investigated binding of AAI to the active site of the compounds I of human CYP1A1, 1A2 and 3A4. These CYPs *O*-demethylate AAI, but with different effectiveness and contribute efficiently to this reaction (see Fig. 5 in our former study [39]). The estimated free energies of AAI binding together with the reaction group distances are shown in Table 1. The results indicate that CYP1A subfamily enzymes, which are more efficient in AAI oxidation, show higher binding affinity toward the AAI then CYP3A4.

**Table 1:** The predicted binding free energies and distances facilitating O-demethylation of AAI bound in selected CYP complexes

Simulated system	The most stable productive orientations of AAI in the complex with CYP	
	Estimated free energy of binding/kJ mol^−1^	O(comp I)-OCH_3_ Distance/Å^a^
CYP1A1	−29.3	4.44
CYP1A2	−32.0	4.90
CYP3A4	−25.0	3.67

^a^Distance between the carbon in the methoxy group of AAI and oxygen atom on heme iron in the complex of an activated CYP enzyme (compound I) with AAI; see Fig. 5

**Fig. 6:**
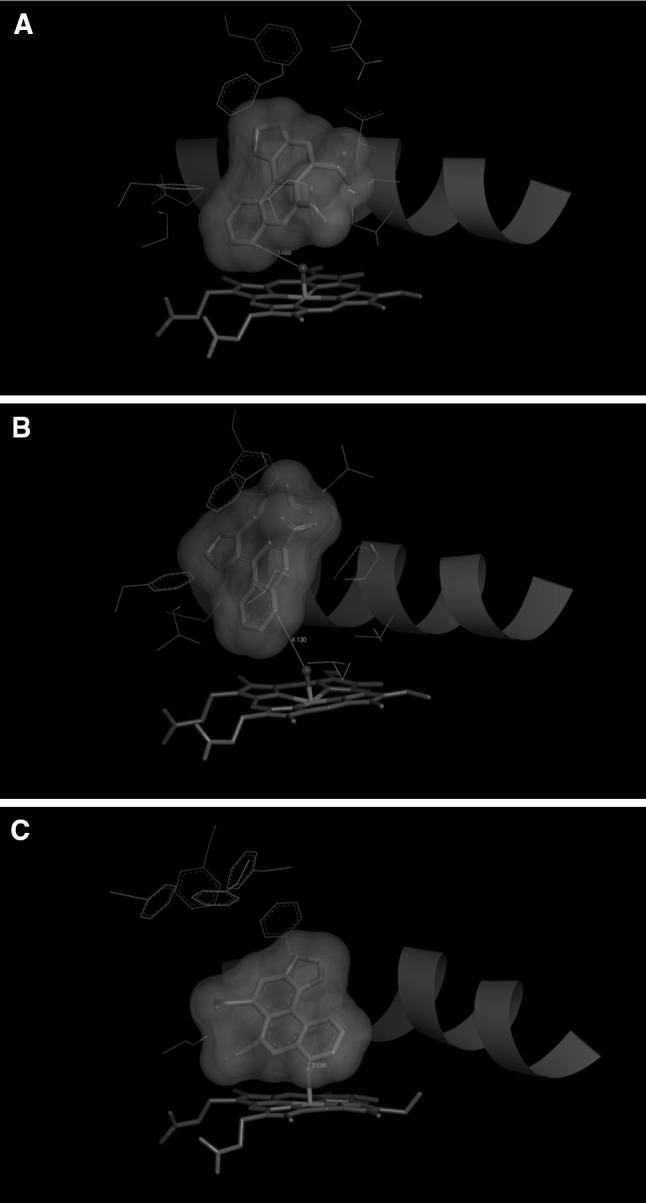
The binding orientations found in molecular docking calculations facilitating C8-hydroxylation of AAII bound in human CYP1A1 (**a**); CYP1A2 (**b**); and CYP3A4 (**c**). AAII, heme and amino acid residues interacting ligand are shown as *bold sticks* and *sticks*, respectively. *Red ribbon* represents a part of the I helix (color figure online)

Now, we evaluated binding of AAII to the same set of enzymes (CYP1A1, 1A2 and 3A4). The estimated free energies of AAII binding together with the reaction group distances are shown in Table 2. The results found in this docking procedure indicate that AAII is best bound to human CYP1A1; however unlike AAI, AAII seems to be a better substrate of CYP3A4 than CYP1A2. It is predicted that CYP3A4 binds AAII more tightly and also in a more suitable position (Table 2; Fig. 6).

**Table 2:** The predicted binding free energies and distances facilitating C8-hydroxylation of AAII bound in selected CYP complexes

Simulated system	The most stable productive orientations of AAII in the complex with CYP	
	Estimated free energy of binding/kJ mol^−1^	O(comp I)-C8 Distance/Å^a^
CYP1A1	−30.9	3.50
CYP1A2	−25.5	4.13
CYP3A4	−27.0	3.70

^a^Distance between the C8 carbon in AAII and oxygen atom on heme iron in the complex of an activated CYP enzyme (compound I) with AAII; see Fig. 5

Small differences in binding free energies between AAI and AAII are insufficient to fully explain the experimental observation that overall oxidation is much lower for AAII than AAI. Nevertheless, the fact that tested CYP enzymes bind the AAI and AAII molecules with similar affinities and that AAII is not metabolized imply that AAII might competitively inhibit AAI oxidation catalyzed by CYPs.

### AAII inhibits the formation of AAIa generated from AAI

To confirm the predicted results on binding of AAII in the active site of tested CYPs, we investigated the effect of AAII on AAIa formation from AAI catalyzed by the microsomal CYP enzyme system. Rat hepatic microsomes were incubated with 0.01 mmol dm^−3^ AAI, both alone and in the presence of AAII (0.001, 0.01 or 0.1 mmol dm^−3^). AAII in this experiment competitively inhibited the formation of AAIa from AAI, with an inhibition constant value (*K*_i_) of 11.3 µM. This finding supports results that were found by flexible *in silico* docking.

### Thermodynamics of AAI and AAII conversion to AAIa

Because the interaction with CYP enzymes was not able to explain a significant difference in AAI and AAII metabolism, we further seek the interpretation in their diverse general amenability to oxidation. The AAIa formation from AAI and AAII proceeds through different reaction mechanisms. O-Demethylation of AAI to AAIa proceeds in two steps: first is α-C-hydroxylation initiated by the attack of the carbon atom of the AAI methoxy group by oxygen originally bound to compound I, which leads to the formation of the α-C-hydroxylated intermediate (step Ia in Fig. 5). This unstable intermediate spontaneously decomposes into AAIa forming formaldehyde as a by-product (step Ib in Fig. 5), while oxidation of AAII proceeded by one-step mono-oxygenation of aromatic carbon 8 (step II in Fig. 5). To test the hypothesis that the observed metabolic difference may originate from the different reaction energetics, we performed ab initio calculations [29] of the reaction steps mentioned above.

The predicted values of reaction free energies (Δ*G* in Table 3) representing individual reaction oxidation steps were predicted using the gas phase ab initio simulation and also using three solvation models: the polarizable conductor calculation model (CPCM), integral equation formalism model (IEFPCM) and the Langevin dipoles model (LD). All reaction steps predicted by these models show negative values of reaction free energies ($\Delta G_{\text{rea}}^{{0^{\prime } }}$); therefore, they are thermodynamically feasible. Interestingly, the predicted relative difference between initial steps (Ia and II) of AAIa formation $(\Delta \Delta G_{{{\text{rea}}{\kern 1pt} ( {\text{AAI}} - {\text{AAII)}}}} )$ always favor AAI over AAII by 9.2–22.6 kJ mol^−1^ (Table 3). In addition, the decomposition of α-C-hydroxylated AAI is also energetically favored; therefore, it further supports AAI metabolism resulting in the overall preference of AAI oxidation by 41–82.8 kJ mol^−1^ over AAII (Table 3). Such significant differences in the reaction free energy Δ*G* of AAIa formation could contribute to the large disparity in hydroxylation potential of AAI and AAII. We propose that this effect can be one of the major reasons why AAI is better oxidized than AAII.

**Table 3:** Standard reaction free energies corresponding to individual reaction steps of AAI and AAII oxidation predicted by quantum chemical approach considering gas phase state and three solvation models CPCM, IEFPCM and LD (for methods, see [29])

Reaction steps (Fig. 5)	$\Delta G_{\text{rea}}^{{0^{\prime } }}$/kJ mol^−1^ AAI → AAIa			$\Delta \Delta G_{{{\text{rea}}{\kern 1pt} ({\text{AAI}} - {\text{AAII}})}}^{{}}$/kJ mol^−1^ AAII → AAIa		
Solvation model	Ia.^a^	Ib.	Ia. + Ib.	II.^a^	Ia. − II.	Ia. + Ib. − II.
Gas phase	−488	−20	−508	−467	−20.5	−41.0
Water (CPCM)	−487	−41	−528	−477	−9.21	−50.6
Water (IEFPCM)	−491	−36	−527	−476	−15.5	−51.9
Water (LD)	−522	−60	−582	−500	−22.6	−82.8

^a^In these steps, free oxygen atom was consider as an oxidant

## Conclusions

The data presented in this study advance our knowledge on the oxidative metabolism of the major components of the natural plant alkaloid and the human carcinogen AA (containing mainly AAI and AAII) by human and rat CYPs and contribute to explain the reasons causing the differences in efficiency in oxidation of these substances to an oxidation metabolite AAIa. Employing rat and human hepatic microsomes containing CYPs and recombinant CYP enzymes, we demonstrated that AAII is oxidized by these enzymes to a much lower extent (if any) than AAI. This phenomenon, found in the present study in in vitro experiments, suggests that AAII is also hardly oxidized in organisms in vivo, being metabolized only by the reductive activation forming AAII–DNA adducts [3, 5, 6]. Indeed, no direct evidence for the formation of AAIa from AAII was found in vivo [17, 18].

The flexible *in silico* docking modeling studies demonstrated almost no differences in binding of AAI and AAII to three of the CYP enzymes that are most effective in AAI oxidation. This finding indicates that both AAs are bound to the active site of CYP-compounds I with similar affinities, which is the first and necessary step for their oxidation. This suggestion was also confirmed by finding that AAII competitively inhibits O-demethylation of AAI to AAIa catalyzed by these enzymes. However, the only AAI is oxidized, whereas essentially no C8-ring hydroxylation of AAII is catalyzed by the CYP systems. These results strongly suggest that binding of AAI and AAII to CYP enzymes is not responsible for differences in AAI and AAII oxidation.

Ab initio calculations employed in this study indicated that the possibility of AAI and AAII being subjected to chemical oxidation differs significantly; the carbon of the methoxy group of AAI is attacked by oxygen (from compound I) forming the unstable α-C-hydroxylated metabolite that is easily decomposed to formaldehyde and AAIa. This decomposition is capable of facilitating the overall production of AAIa from AAI, because it is finally energetically more feasible than the C8-ring hydroxylation of AAII. Thus, these results demonstrate that the key factor causing the differences in AAI and AAII oxidation is their different amenability to oxidation.

## Experimental

Aristolochic acid mixture (AA, 38% AAI, 58% AAII) and NADPH (as tetrasodium salt; ~98% purity) were purchased from Sigma Chemical Co. (St Louis, MO, USA). AAI (CAS Number 313-67-7) and AAII (CAS Number 475-80-9) were purified from the commercially available AA mixture by reverse-phase chromatography as described previously [23].

### Animal experiments and isolation of hepatic microsomes

All animal experiments were conducted in accordance with the Regulations for the Care and Use of Laboratory Animals (311/1997, Ministry of Agriculture, Czech Republic), which is in compliance with the Declaration of Helsinki. Male Wistar rats (~125–150 g, AnLab, Czech Republic) placed in cages in temperature- and humidity-controlled rooms were acclimatized for 5 days and maintained at 22 °C with a 12 h light/dark period. Standardized diet (ST-1 diet from Velaz, Czech Republic) and water were provided ad libitum. Rats were treated with inducers of CYP1A (Sudan I), CYP2B (PB), ethanol (CYP2E1) and CYP3A (PCN) as follows: (1) Ten 5-week-old male Wistar rats (~125–150 g) were injected i.p. with 20 mg kg^−1^ b.w. Sudan I in maize oil once a day for three consecutive days as reported previously [38]. Animals in the control group received the same volume of maize oil on the 3 days. Rats were killed 24 h after the last treatment by cervical dislocation. (2) Ten 5-week-old male Wistar rats (~125–150 g) were pretreated with PB (0.1% in drinking water for 6 days) as described previously [38]. Animals in the control group received drinking water. Rats were killed after treatment by cervical dislocation. (3) Ten 5-week-old male Wistar rats (~125–150 g) were pretreated with ethanol (10% in drinking water for 7 days) as described previously [43]. Animals in the control group received drinking water. Rats were killed after treatment by cervical dislocation. (4) Ten 5-week-old male Wistar rats (~125–150 g) were injected i.p. with 50 mg kg^−1^ b.w. PCN dissolved in maize oil for four consecutive days as reported previously [44]. Animals in the control group received the same volume of maize oil. Rats were killed 24 h after the last treatment by cervical dislocation. For all treatment groups, livers of the animals were removed immediately after killing, frozen in liquid nitrogen and stored at −80 °C until isolation of microsomal fractions. Pooled microsomes were prepared from ten rat livers/group as reported [25] and used for experiments of our present study. As the control microsomes, those from rats treated with 1 cm^3^ of sunflower oil (by gavage, see above) were utilized. The activities of the CYP marker substrates in these control microsomes did not differ significantly from those in other control microsomes. Microsomal fractions were stored at −80 °C until analysis. Protein concentrations in the microsomal fractions were assessed using the bicinchoninic acid protein assay with bovine serum albumin as a standard [45].

### AAIa formation by rat and human hepatic microsomes and Supersomes™

The incubation mixtures, in a final volume of 0.250 cm^3^, consisted of 100 mmol dm^−3^ potassium phosphate buffer (pH 7.4), 1 mmol dm^−3^ NADPH, 1 mg human or rat hepatic microsomal protein and 0.01 mmol dm^−3^ AAI or AAII or 0.02 mmol dm^−3^ AA. Incubations with microsomes were carried out at 37°C for 20 min (AAI oxidation to AAIa was linear up to 25 min [33, 38]. Control incubations were carried out (1) without microsomes, (2) without NADPH or (3) without AAI, AAII or AA. Human hepatic microsomes (male and female) and Supersomes™, microsomes isolated from insect cells transfected with baculovirus constructs containing cDNA of single rat CYPs (CYP1A1, CYP1A2, CYP2A1, CYP2A2, CYP2B1, CYP2C6, CYP2C11, CYP2C12, CYP2C13, CYP2D1, CYP2D2, CYP2E1, CYP3A1 and CYP3A2) or of single human CYPs (CYP1A1, CYP1A2, CYP2A6, CYP2B6, CYP2C9, CYP2C19, CYP2D6, CYP2E1, CYP3A4) and expressing POR and/or cytochrome *b*_*5*_, were obtained from Gentest Corp and tested for their efficiencies to oxidize AA. Incubation mixtures in a final volume of 0.250 cm^3^ consisted of 100 mmol dm^−3^ potassium phosphate buffer (pH 7.4), 1 mmol dm^−3^ NADPH, 50 nmol dm^−3^ CYPs in Supersomes™ and 0.01 mmol dm^−3^ AAI or AAII. AA metabolites including AAIa were analyzed by high-performance liquid chromatography (HPLC) as described below and in the previous studies [33, 36, 38].

### Inhibition studies

Incubation mixtures, in a final volume of 0.250 cm^3^, consisted of 100 mmol dm^−3^ potassium phosphate buffer (pH 7.4), 1 mmol dm^−3^ NADPH, 1 mg rat hepatic microsomal protein and 0.01 mmol dm^−3^ AAI without or with 0.001, 0.01 or 0.1 mmol dm^−3^ AAII. Mixtures were incubated at 37 °C for 25 min. Formation of AAIa was analyzed by HPLC [33, 36, 38]. The value of the inhibition constant K_i_ for AAII was determined by the Dixon plot [46].

### HPLC analysis of AAIa formation

AA, AAI or AAII and their metabolites (including AAIa) were extracted from incubations with ethyl acetate (2 × 1 cm^3^), the extracts were evaporated to dryness and the residues redissolved in 0.03 cm^3^ of methanol and subjected to reverse-phase HPLC. HPLC was performed with a reversed-phase column (Nucleosil 100-5 C_18_, 25 × 4.0 mm, 5 mm; Macherey-Nagel) preceded by a C-18 guard column, using a linear gradient of acetonitrile (20–60% acetonitrile in 55 min) in 100 mmol dm^−3^ triethylammonium acetate with a flow rate of 0.5 mmol dm^−3^ min^−1^. A Dionex HPLC pump P580 with UV/VIS UVD 170S/340S spectrophotometer detector was set at 250 nm and a CHROMELEON™ 6.01 integrator was used for the integration of peaks. A peak eluting at retention time (r.t.) 22.1 (22.7) min was identified as AAIa using mass spectroscopy analysis [38].

### Molecular docking of AAI and AAII into compounds I of human CYP1A1, 1A2 and 3A4

The X-ray based coordinates of human CYP1A1 (2.6 Å resolution, PDB ID 4I8V) [47], human CYP1A2 (1.95 Å resolution, PDB ID 2HI4) [48] and CYP3A4 (2.74 Å resolution, PDB ID 1W0G) were used as starting structures for modeling of AAI or AAII interactions with the ground state of CYP enzymes. During structure preparation, hydrogen atoms were added and crystallographic water and ligand molecules were removed, and the usual protonation states and Gasteiger partial charges were assigned to all residues, except for the atomic charge of the ferric ion of the heme cofactor, for which a value more consistent with a metal in octahedral coordination was used [49]. The geometries and charges of a ligands (AAI and AAII) were predicted using ab initio calculations on the Hartree–Fock level of theory in conjunction with the 6-31 + G(d) basis set. These ab initio calculations were performed with program Gaussian 03 [50].

We employed a hybrid global–local Lamarckian genetic algorithm implemented in Autodock v4.2.6 program [51] suite to evaluate binding free energies and preferred binding modes for studied compounds. All rotatable bonds of the ligands and 10–11 selected amino acid side chains, CYP1A1 (S122, F123, N221, F224, F258, D313, D320, T321, V382, L496, T497), CYP1A2 (T124, F125, T223, F226, F260, D313, D320, T321, L382, L497, T498) and CYP3A4 (F108, S119, F213, F215, F241, F304), were allowed to rotate freely. We performed an extensive search (5000 docking runs per system) of the most preferred binding modes of an AAI molecule within a 57 × 47 × 47 grid box centered on the substrate binding cavity. Similar resulting structures (RMSD lower than 2.0 Å) were grouped and finally sorted by binding free energy of the best binding structure within each cluster. As a result, a set of binding modes with similar binding energies was obtained for every system. We assume that only the orientations with a sufficiently short distance between carbon of the methoxy group of AAI or the C8 carbon atom of AAII and the activated oxygen atom in the CYP compound I would facilitate the AAI or AAII oxidation.

### Quantum chemical calculation

The geometry optimizations of all reactants and products were done using ab initio approach implemented in Gaussian09 program suite [52]. All calculations were performed on the Hartree–Fock (HF) level of theory in conjunction with 6-31+G(d) basis set. The thermal corrected Gibbs free energies were in all models obtained from electronic calculations and harmonic vibration frequencies of these optimized structures. The reaction Gibbs free energies of individual reaction steps evaluated here were calculated as the total free energies of products minus the total free energies of reactants. Initially, the geometry optimization was performed without considering the solvent (in gas phase), and then solvent effect was estimated by performing energy optimizations using the polarizable conductor calculation model (CPCM) [53], integral equation formalism model (IEFPCM) [52] with default atomic radii. The solvation free energy of the considered compounds was also predicted using Langevin dipole model (LD) with the ChemSol program v2.1 [53]. Merz–Kollman partial atomic charges obtained from ab initio calculations served as an input for these LD calculations.

### Statistical analyses

For statistical data analysis, we used Student’s *t* test. All *P* values are two-tailed and considered significant at the 0.05 level.
